# Sarcopenia associates with SNAP-25 SNPs and a miRNAs profile which is modulated by structured rehabilitation treatment

**DOI:** 10.1186/s12967-021-02989-x

**Published:** 2021-07-21

**Authors:** Simone Agostini, Roberta Mancuso, Andrea Saul Costa, Franca Rosa Guerini, Fabio Trecate, Rossella Miglioli, Elisabetta Menna, Beatrice Arosio, Mario Clerici, the SA. M. B. A. project

**Affiliations:** 1 IRCCS Fondazione Don Carlo Gnocchi ONLUS, P.zza Morandi, 3, 20100 Milan, Italy; 2 CNR-Institute of Neuroscience, Milan, Italy; 3 Humanitas Clinical and Research Center–IRCCS, via Manzoni 56, 20089 Rozzano, MI Italy; 4 Geriatric Unit, Fondazione IRCCS Ca’ Granda Ospedale Maggiore Policlinico, Milan, Italy; 5 Department of Clinical Sciences and Community Health, University of Milan, Milan, Italy; 6 Department of Pathophysiology and Transplantation, University of Milan, Milan, Italy

**Keywords:** Sarcopenia, Rehabilitation, SNAP-25, miRNAs, Biomarkers

## Abstract

**Background:**

Sarcopenia is a loss of muscle mass and strength causing disability, morbidity, and mortality in older adults, which is characterized by alterations of the neuromuscular junctions (NMJs). SNAP-25 is essential for the maintenance of NMJ integrity, and the expression of this protein was shown to be modulated by the *SNAP-25* rs363050 polymorphism and by a number of miRNAs.

**Methods:**

We analysed these parameters in a cohort of sarcopenic patients undergoing structured rehabilitation. The rs363050 genotype frequency distribution was analyzed in 177 sarcopenic patients and 181 healthy controls (HC). The concentration of seven miRNAs (miR-451a, miR-425-5p, miR155-5p, miR-421-3p, miR-495-3p, miR-744-5p and miR-93-5p), identified by mouse brain miRNome analysis to be differentially expressed in wild type compared to *SNAP-25*^±^ heterozygous mice, was analyzed as well by droplet digital PCR (ddPCR) in a subgroup of severe sarcopenic patients undergoing rehabilitation.

**Results:**

The *SNAP-25* rs363050 AA genotype was significantly more common in sarcopenic patients compared to HC (p_c_ = 0.01); miR-451a was significantly up-regulated in these patients before rehabilitation. Rehabilitation modified miRNAs expression, as miR-155-5p, miR-421-3p, miR-451a, miR-425-5p, miR-744-5p and miR-93-5p expression was significantly up-regulated (p < 0.01), whereas that of miR-495-3p was significantly down-regulated (p < 0.001) by rehabilitation. Notably, rehabilitation-associated improvement of the muscle-skeletal SPPB score was significantly associated with the reduction of miR-451a expression.

**Conclusion:**

These results support the hypothesis of a role for SNAP-25 in sarcopenia and suggest SNAP-25-associated miRNAs as circulatory biomarkers of rehabilitative outcome for sarcopenia.

**Supplementary Information:**

The online version contains supplementary material available at 10.1186/s12967-021-02989-x.

## Background

In the last 50 years human life expectancy has greatly increased. This is a real-life improvement that, on the converse, has also caused important socio-demographic issues due to the rapid aging of the population, and has resulted in a dramatic increase in the prevalence of chronic pathologies. Sarcopenia, in particular, the age-dependent loss of muscle mass and function, is a common condition among older persons, and is associated with several adverse health outcomes causing disability, morbidity, and mortality [1–3].

The core of sarcopenia involves quantitative and qualitative loss of skeletal muscle as well as alterations of neuromuscular junctions (NMJs), with a predominance of type I fibers that leads to atrophy of type II fibers [4], and accumulation of adipose tissue around and between muscle fibers [5]. Because the muscle contractile activity is regulated by the central nervous system at the NMJs, and NMJs are fundamental for the maintenance of the integrity of motor nerve and muscle fibers [6], sarcopenia was hypothesized to have a neurological origin [7]. The structural features of NMJ are similar to those of other chemical synapses, and many of the molecules involved in the formation of the NMJ have also an essential role in its maintenance, such as the SNARE complex [8, 9]. The core SNARE synaptosomal-associated protein of 25 kDa (SNAP-25), in particular, could be a key factor in sarcopenia, as it accumulates in the plasma membrane of nerve terminals at NMJ, consistently with its role in regulating exocytosis at peripheral and central synapses [10, 11]. To note, adult *SNAP-25*^±^ mice expressing low SNAP-25 protein level, were shown to be characterized by an impaired grip strength [12].

SNAP-25 expression is modulated by the rs363050 polymorphism, which is localized in intron 1 of the gene encoding for *SNAP-25*, thus, GG homozygosis resulted in a reduced gene expression compared to the AA homozygosis [13]. As the majority of genes, *SNAP-25* expression is also regulated by microRNAs (miRNAs), short non-coding RNAs involved in mRNA silencing and post-transcriptional modulation of gene expression [14]. Of note, the differential expression of SNAP-25-related miRNAs as well as polymorphisms in the *SNAP-25* gene (i.e. rs363050), which may result in the modulation of SNAP-25 expression, were suggested to play a pathogenic role in different brain diseases including Alzheimer's Disease, Parkinson's Disease and Amyotrophic Lateral Sclerosis [15, 16], but also Attention Deficit Hyperactivity Disorder (ADHD) [17–21] and early-onset bipolar disorders [22]. Whether these mechanisms can be involved in sarcopenia is not known. Indeed, even if research on sarcopenia prevention and treatment is developing quickly, many questions are still unanswered. Furthermore, physical exercise was shown to influence miRNA expression [23, 24], however to our knowledge no evidence has been reported regarding the impact of structured physical rehabilitation programs on miRNA expression in sarcopenic patients. Here we analysed the possible association of rs3603050 *SNAP-25* polymorphism with sarcopenia, whether miRNAs related with the expression of this gene are differentially expressed in sarcopenic patients, and, finally, the possible effect of rehabilitative treatment on miRNAs expression.

## Material and methods

### Patients and controls

*SNAP-25* genotyping was performed in three hundred fifty-eight Caucasian individuals: 177 patients with a diagnosis of sarcopenia (35 male and 142 female) and 181 sex and age matched HC (49 male and 132 female) (Table 1). Subjects were recruited by the Palazzolo Institute, Fondazione Don Carlo Gnocch ONLUSi, and by the Fondazione IRCCS Ca’ Granda, Ospedale Maggiore Policlinico, both in Milan, Italy. Patients were diagnosed as sarcopenic according to the European Working Group on Sarcopenia in Older People (EWGSOP) [2].

**Table 1:** Demographic and clinical characteristics of the individuals enrolled in the study

	Sarcopenic patients	Healthy controls
N	177	181
Gender (M:F)	35:142	49:132
Age, years	77.1 ± 6.5	78.5 ± 9.0
MMSE	28.3 ± 1.9	29.5 ± 0.4
SPPB	2 1–6	11* 10–12
Right Handgrip	19.4 ± 7.6	–
Left Handgrip	16.6 ± 6.6	–

Data for Age and MMSE are reported as mean ± standard deviation, median and Interquartile range are reported for SPPB values

*MMSE* mini mental state evaluation, *SPPB* short physical performance battery

^*^ p < 0.0001

At the time of recruitment, patients underwent a comprehensive geriatric multidimensional evaluation that included lower extremity function evaluation with the Short Physical Performance Battery (SPPB) [25], cognitive function evaluation with the Mini-Mental State Examination (MMSE) [26] and the Clock Drawing test (CDT) (score range 0–5) [27]. The emotional status was evaluated using the Yesavage Geriatric Depression Scale, which consists of 30 questions and is scored between 0 and 30 points [28]. Comorbidity was evaluated with the Charlson Comorbidity Index (CCI) [29]. Finally, the functional status was assessed using the ADL (activity of daily living or KATZ index) (bathing, dressing, toileting, transfer, feeding, continence) and the IADL (Lawton-Brody instrumental activity of daily living) (telephone use, housekeeping, laundry, medication use, transportation, preparing meal, shopping, handling finances) tests. ADL and IADL scales are scored between 0 to 6 and 0 to 8 points, respectively [30, 31].

Subjects with MMSE < 24, undergoing steroid therapy, that were clinically instable, had a concomitant diagnosis of neoplastic or neurodegenerative diseases, or were unable to participate safely in the intervention program, were excluded by the study.

miRNA expression was evaluated in a sub-group of hospitalized severe sarcopenic patients (SPPB < 3, n = 45) who were undergoing a 30-days structured rehabilitation treatment as follows: twice day session of 40’ in the morning and 30’ in the afternoon with assisted mobilization, progressive muscle strengthening, associated with progressivity of the load, standing work proprioceptive postural balance, walking training firstly with an assisted way and then without. For each subject, SPPB was calculated before and after the rehabilitative treatment. A frailty index was computed following the criteria described by Searle et al. [32] and taking into account a wide range of age-related signs, symptoms, disabilities, and diseases. HC were age-and-sex-matched individuals without sarcopenia who had undergone surgery treatment for knee replacement or hip/knee fractures (n = 44). The study conformed to the ethical principles of the Declaration of Helsinki; all subjects gave informed and written consent according to a protocol approved by the ethics committee of IRCCS Fondazione Don Carlo Gnocchi ONLUS (n#9_04/04/2018).

### Mouse brain miRNome analyses

All experiments followed the guidelines established by the European Directive 2010/63/EU and the Italian Governing Law 26/2014 (PR-09/2013). All efforts were made to minimize the number of subjects used and their suffering. SNAP-25 wild-type and *SNAP-25* heterozygous mice were housed in cages with free access to food and water at 22 °C and with a 12-h alternating light/dark cycle. Genotyping was performed by PCR as described in Washbourne et al. [33]. *SNAP25*^+/+^ and *SNAP25*^±^ brains (12 months old mice) have been collected and stored at -80 °C until use.

miRNome analyses were performed on 20 mg brain samples of 8 female mice (4 wild type and 4 *SNAP-25 ±* heterozygous mice) by qPCR by Qiagen Genomic Services (Qiagen GmbH, Hilden, Germany). Briefly, after RNA extraction and the reverse transcription into cDNA, the miRCURY LNA miRNA PCR mouse and Rat, panel I (372 miRNAs analyzed, Qiagen) was used for the analysis. miRNA expression data were normalized using the global mean method.

### *SNAP-25* genotyping

Whole blood was collected for all the subjects and genomic DNA was isolated by phenol–chloroform extraction. Taqman SNP Genotyping Assay (Life Technologies, Foster City, CA, US) was used to type SNP rs363050 of *SNAP-25* [34].

### Serum miRNA isolation and cDNA reverse transcription

For severe sarcopenic patients also serum was obtained from peripheral blood by centrifugation at 1800*g* for 10 min. The absence of hemolysis in serum was evaluated by visual inspection and by spectrophotometric measurement of absorbance of hemoglobin at 414 nm [35]. miRNA isolation from 200 μl of serum was semi-automatically performed with a column-based kit (MiRNeasy serum/plasma kit, Qiagen GmbH, Hilden, Germany) by Qiacube (Qiagen GmbH, Hilden, Germany), according to manufacturer's protocol. After quantitation by Qubit (Qiagen GmbH, Hilden, Germany), equal concentration of extracted miRNAs was retro-transcribed in cDNA (miRCURY LNA RT kit, Qiagen GmbH, Hilden, Germany) for all samples. To avoid variations due to sample differences and handling, all the variable involved in the procedure were kept consistent throughout the study.

### Quantification of selected circulatory miRNA

miRNA quantitation was performed by droplet digital PCR (ddPCR QX200, Bio-Rad, Hercules, CA, US). Briefly, 3 µl of diluted cDNA (1:25) was mixed with LNATM-specific primers (Qiagen GmbH, Hilden, Germany), and ddPCR EvaGreen Supermix (Bio-Rad, Hercules, CA, US), which was then emulsified with droplet generator oil (Bio-Rad, Hercules, CA, US) using a QX200 droplet generator, according to the manufacturer’s instruction. The droplets were then transferred to a 96-well reaction plate and heat-sealed with a pierceable sealing foil sheet (PX1, PCR plate sealer, Bio-Rad, Hercules, CA, US). PCR amplification was performed in sealed 96-well plate using a T100 thermal cycler (Bio-Rad, Hercules, CA, US) as follows: 10 min at 95 °C, 40 cycles at 94 °C for 30-s and at 58° for 60 s, followed by 10 min at 98 °C and a hold at 4 °C. The 96-well plate was then transferred to a QX200 droplet reader (Bio-Rad, Hercules, CA, US). Each well was queried for fluorescence to determine the quantity of positive events (droplets), and the results were displayed as dot plots. The miRNA concentration was expressed as copies/ng of extracted RNA.

### Statistical analysis

Normally distributed data were expressed as mean ± standard deviation, and comparisons among groups were analyzed by ANOVA test and Student t-test, when appropriate. Not-normally distributed data were expressed as median and interquartile range (IQR: 25th and 75th percentile), and comparisons were analyzed by Kruskal–Wallis and Mann–Whitney U test, as appropriate, and with Wilcoxon signed-rank test for paired data. Correlations were analyzed using Spearman's correlation coefficient and multiple logistic regression analysis. p-values corresponding to ≤ 0.05 were statistically significant. Qualitative data were compared using Chi-squared test and p-value was considered significant when ≤ 0.05 after Bonferroni correction for degrees of freedom (pc) in 2 × 3 ad 2 × 2 contingency tables. Bioinformatics analysis was conducted by applying the Web-based tool miRSystem [36] to identify the shared pathways targeted by the selected miRNAs. The statistical analyses were accomplished using commercial software (MedCalc Statistical Software version 14.10.2, Ostend, Belgium).

## Results

### Clinical parameters

Demographic and clinical characteristics of the study population are summarized in Table 1. Gender, age and Mini Mental State Evaluation (MMSE) were similar in the two groups. Regarding comorbidity in sarcopenic patients: 35/45 had a diagnosis of hypertension, 19/45 cardio-vascular diseases, 12/45 osteoporosis, 10/45 diabetes, 9/45 arthrosis, 7/45 thyroiditis, 4/45 chronic obstructive pulmonary disease (COPD), 2/45 asthma and 1/45 rheumatic disease. The individual comorbidity for each sarcopenic patient is reported at Additional File 1.

As expected, the median of SPPB was significantly lower in sarcopenic patients (median and Interquartile range: 2; 1–6) compared to HC (11; 10–12) (p < 0.0001). Table 2 summarizes the demographic and clinical characteristics of the subgroup of the severely sarcopenic patients who underwent rehabilitation, and of the age-and-sex-matched HC; circulatory miRNA expression was analyzed in all these individuals. As expected, median SPPB score of the sarcopenic patients increased after the rehabilitative treatment (median and Interquartile range: 2; 1–3) compared to the values observed before rehabilitation (0; 0–1) (p = 0.002). The majority of the subjects presents more than one comorbidity (Additional file 1: Table S1), probably due to the age of enrolled subjects [37]. The mean ± standard deviation Frailty Index of these patients is 0.19 ± 0.13.

**Table 2:** Demographic and clinical characteristics of the severely sarcopenic patients who underwent rehabilitation and in whom circulatory miRNAs were analyzed and of the age-and-sex-matched non-sarcopenic healthy controls (HC)

	Sarcopenic patients	Healthy controls
N	45	44
Gender (F:M)	37:8	32:12
Age, years	73.3 ± 8.3	71.9 ± 7.1
MMSE	27.8 ± 2.4	29.4 ± 0.5
SPPB pre-rehabilitation	0 0–1	11* 10–12
SPPB post-rehabilitation	2.2 1.2–3.0**	–
Right Handgrip pre-rehabilitation	19.4 ± 7.6	–
Right Handgrip post-rehabilitation	20.2 ± 7.8	–
Left Handgrip pre-rehabilitation	16.6 ± 6.6	–
Left Handgrip post-rehabilitation	16.1 ± 7.5	–

Data for Age and MMSE are reported as mean ± standard deviation, median and Interquartile range are reported for SPPB values

*MMSE* mini mental state evaluation, *SPPB* short physical performance battery

^*^ p = 0.002

^**^ p < 0.0001 pre-vs*.* post-rehabilitation SPPB score

### *SNAP-25* rs363050 genotyping

Initial molecular genotyping analyses showed that the *SNAP-25* (A/G) rs363050 polymorphism genotype distribution was in Hardy–Weinberg (HW) equilibrium. Further analyses showed that the overall genotype distribution of *SNAP-25* rs363050 was significantly different in sarcopenic patients compared to HC (p = 0.001; d.f.: 2; χ^2^ = 12.94). In particular, the *SNAP-25* rs363050 AA genotype resulted to be more frequent in sarcopenic patients (40%) compared to HC (27%) (p_c_ = 0.01; OR = 1.85, 95% CI 1.19–2.90) (Table 3). These differences were not confirmed when analyses were limited to the subgroup of severely diseased patients who were undergoing rehabilitation and in whom circulatory miRNAs were analyzed, probably due to the relatively small sample size (Table 3).

**Table 3:** *SNAP-25* rs353050 genotype distribution of the individuals enrolled in the study (overall and severely sarcopenic patient undergoing rehabilitation in which circulatory miRNAs were analyzed)

*SNAP-25* rs363050*	Sarcopenic patients	Healthy controls
Overall group	n. 177	n. 181
AA^**^	71 (40%)	48 (27%)
AG	73 (41%)	109 (60%)
GG	33 (19%)	24 (13%)
Severely sarcopenic patients undergoing rehabilitation	n. 45	n. 44
AA	17 (38%)	15 (33%)
AG	22 (49%)	25 (57%)
GG	6 (13%)	4 (10%)

Genotype distribution is reported as absolute number and percentage of subjects

^*^ p = 0.001; d.f.: 2; χ^2^ = 12.94;

^**^ p_c_ = 0.01; OR = 1.85; 95% CI 1.19–2.90

### Mouse brain miRNome profiling according to *SNAP-25* genotyping

MiRNome expression profile of mouse brain identified in all samples 231 miRNAs, with an average of 258 miRNAs detectable per sample. Among these miRNAs, 2 were significantly deregulated in wild type compared to *SNAP-25*^±^ heterozygous mice: miR-451a and miR-425-5p. Five other miRNAs were differentially expressed as well, as their expression ratio was > 2.5-fold or < − 2.5-fold: miR155-5p, miR-421-3p; miR-495-3p, miR-744-5p and miR-93-5p (data not shown). For these reasons, and because all these miRNAs are homologous between mouse and human, the expression of these 7 miRNAs was analyzed in sera of sarcopenic patients and HC.

### miRNAs expression in association with *SNAP-25* rs363050 genotypes

**Fig. 1:**
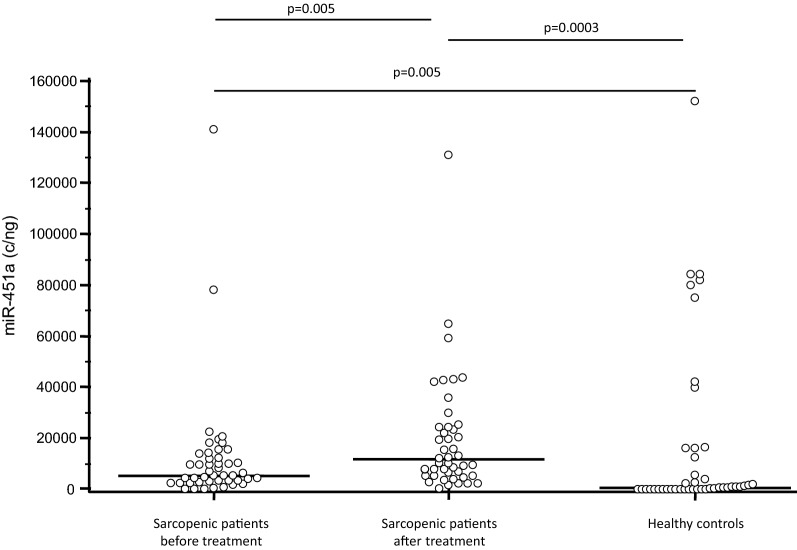
miR-451a expression (copies/ng) in serum of sarcopenic patients before and after rehabilitative treatment and age-and-sex-matched healthy controls. Horizontal bars indicate medians

Before initiation of the rehabilitation protocol, miR-451a expression was significantly higher in sarcopenic patients compared to HC (p = 0.005) (Fig. 1); the expression of the other 6 miRNAs was similar in the two groups.

**Fig. 2:**
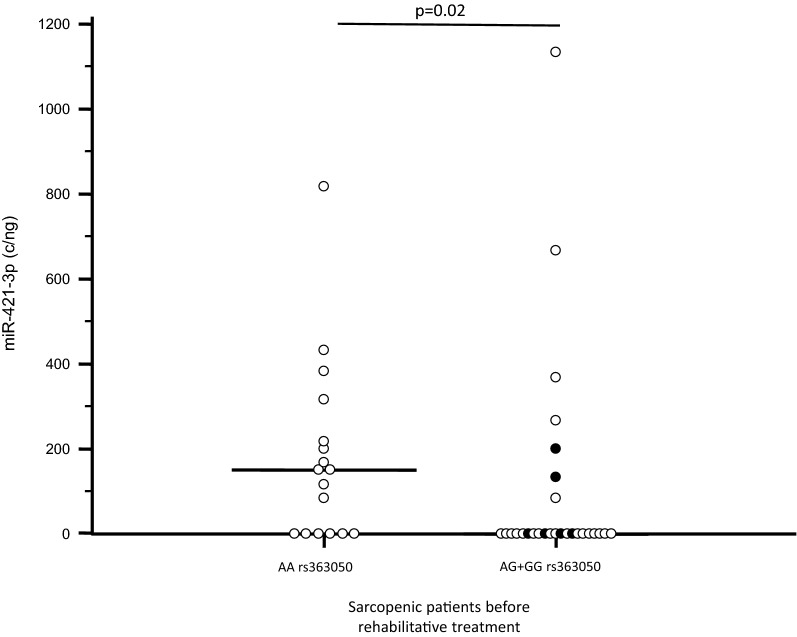
miR-421-3p expression (copies/ng) in serum of sarcopenic patients before rehabilitative treatment. Results are divided according to *SNAP-25 *rs363050 categorization (AA vs. AG + GG; black dots represent GG genotype). Horizontal bars indicate medians

Upon dividing miRNAs results based on the *SNAP-25* rs363050 genotype, results showed that miR-421-3p expression was increased in *SNAP-25* AA genotype homozygous (median and Interquartile range: 150; 0–241 copies/ng) compared to AG heterozygous (0; 0–0 copies/ng) (p = 0.03), and rs363050 GG homozygous (0; 0–133 copies/ng) sarcopenic patients before rehabilitation. The statistical differences remained even when patients that were homozygote for the major allele rs363050 AA were compared with those carrying the minor allele both as homozygous and heterozygous rs363050 genotypes (AG + GG) (150; 0–241 vs. 0; 0–41.77 copies/ng; p = 0.02) (Fig. 2). Interestingly, these differences disappeared at the end of rehabilitation. No differences were observed for the other 6 miRNAs in association with *SNAP-25* rs363050 genotype. No differences emerged when male and female were analyzed separately (data not shown).

### Effects of rehabilitation on miRNAs expression

The expression of all the analyzed miRNAs was modified by the 30-days rehabilitative treatment. Thus, 6 of these miRNAs (miR-155-5p, miR-421-3p, miR-451a and miR-425-5p, miR-744-5p and miR-93-5p) were significantly up-regulated (p < 0.01 for all miRNAs), whereas mir-495-3p was significantly down-regulated (p < 0.001) after treatment. Table 4 summarizes the miRNAs concentration in enrolled population.

**Table 4:** Absolute miRNA concentration measured by ddPCR in serum of the severely sarcopenic patients undergoing rehabilitation and healthy controls

	Sarcopenic patients before rehabilitation	Sarcopenic patients after rehabilitation	Healthy controls
miR-93-5p (copies/ng)	183.00; 83.30–429.00#	617.00; 267.00–1320.00	117.00; 0.00–692.00^^^
miR-155-5p (copies/ng)	0.00; 0.00–121.00#	117.00; 100.00–292.00	53.30; 0.00–117.00^^^
miR-421-3p (copies/ng)	0.00; 0.00–175.00#	233.00; 158.00–454.00	66.7; 0.00–183.00^#^
miR-425-5p (copies/ng)	41.7; 17.10–97.90#	333.00; 104.00–1110.00	95.00; 0.00–433.00^^^
miR-451a (copies/ng)	5420.00; 2820.00–12,600.00^	12,233.00; 5154.17–30,041.67	983.00; 0.00–17,900.00*
miR-495-3p (copies/ng)	150.00; 0.00–271.00#	0.00; 0.00–150.00	150.00; 83.30–242.00^^^
miR-744-5p (copies/ng)	0.00; 0.00–187.00#	883.00; 0.00–1920.00	0.00; 0.00–92.52^^^

Data are reported as median and interquartile range

^#^ p < 0.001 HC vs. sarcopenic patients after rehabilitation

^^^ p < 0.01 HC vs. sarcopenic patients after rehabilitation

^*^ p < 0.01 HC vs. sarcopenic patients before and after rehabilitation

**Fig. 3:**
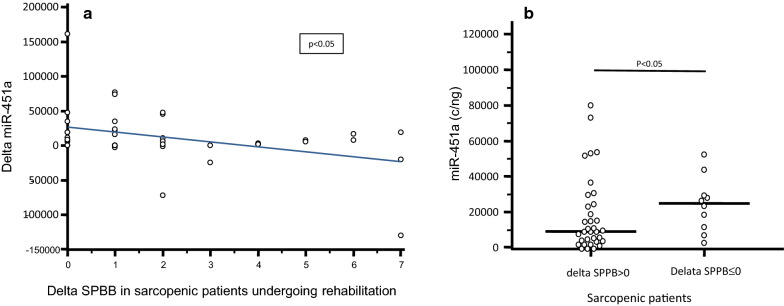
**a** Correlation between the change (delta) of SPPB and miR-451a expression in serum of sarcopenic patients. **b** miR-451a expression (copies/ng) in serum of sarcopenic patients before and after rehabilitative treatment in whom rehabilitation was (delta SPPB > 0) or was not (delta SBBP ≤ 0) successful. Horizontal bars represent medians

Finally, when the difference of the expression before and after rehabilitative treatment (delta) was calculated for miRNAs in relationship with SPPB, a significant negative correlation was found between the delta of miR-451a expression and the delta of SPPB (p < 0.05) (Fig. 3a). This suggests that when, after rehabilitation, the SPPB score increases, miR-451a decreases more, approaching the values observed in healthy controls. This association between miR-451a expression change after rehabilitation and rehabilitative outcome was confirmed also after multiple logistic regression analysis adjusted for age and frailty index (p = 0.05). Confirming this hypothesis, miR-451a expression after rehabilitation was significantly lower in patients with a positive variation of physical performance (delta SPPB > 0; n = 34) compared to those with no or negative variation (SPPB ≤ 0) (p < 0.05; n = 11) (Fig. 3b). Importantly, the frailty index was not significantly different between the two groups (delta SPPB > 0: 0.14; 0.14–0.29; delta SPPB ≤ 0: 0.29; 0.12–0.32). Results were similar when analyses were performed in patients and controls after splitting the two groups according to gender (data not shown).

### Bioinformatics analysis

**Fig. 4:**
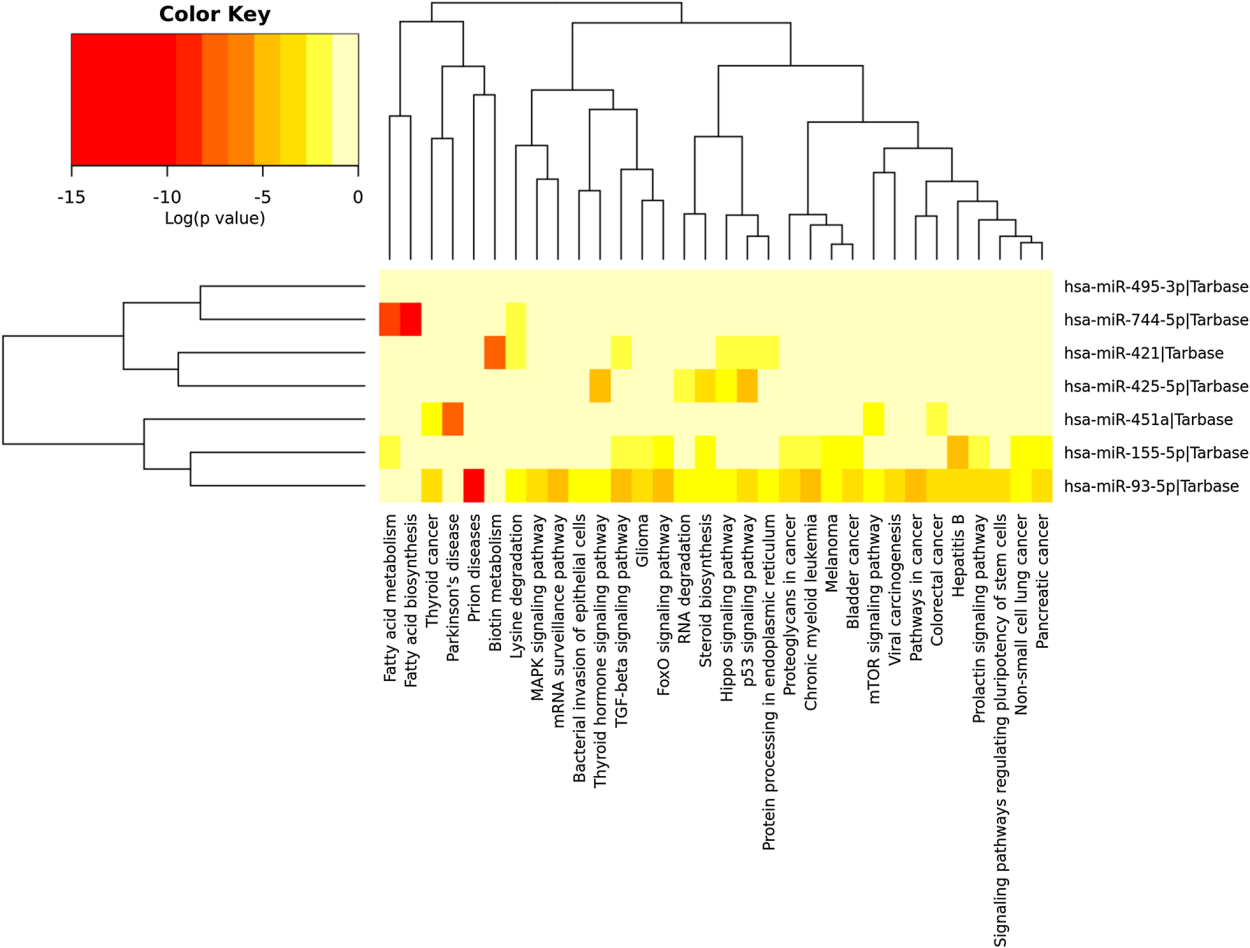
Hierarchical cluster and miRNA/KEGG pathways heat map among 7 miRNAs deregulated in brain of *SNAP-25* ± vs. wild type mice. The map was obtained using the Diana tools miRPath v.3 software

Bioinformatics analysis showed that the analyzed miRNAs target human genes pathways involved in fatty acid metabolism/biosynthesis (p < 0.0001), steroid biosynthesis, and biotin metabolism (Fig. 4). To note, four of the human pathways (MAPK, FoxO, TGFβ and mTOR signalling pathways) were indicated by bioinformatic analysis were previously indicated involved in sarcopenia [38]. Additionally, as most miRNAs have been analyzed in the scenario of oncology research, most pathways show weak association related to cancer.

## Discussion

Sarcopenia is a multifactorial and complex disease in which several genes and proteins contribute to the decline of muscle mass and functions. In this study we focused our attention on *SNAP-25*, a gene whose polymorphisms have been shown to be associated with a number of pathologies, including Alzheimer’s and Parkinson’s diseases, schizophrenia, attention deficit hyperactivity disorders and epilepsy; our results indicate that *SNAP-25* SNPs also play a role in sarcopenia. Thus, our results for the first time show that sarcopenia is associated with *SNAP-25* polymorphisms and with a particular profile of miRNAs that regulate SNAP-25 expression. Notably, data herein also indicate that successful muscle-skeletal rehabilitation outcome correlates with modifications in miRNAs expression, suggesting that miRNAs expression could be used as a biomarker for sarcopenia.

The SNAP-25 protein is a component of the multiproteic SNARE complex and is responsible for the fusion of synaptic vesicles with the plasma membrane during exocytosis, playing a significant role in the maintenance of the NMJ integrity and functionality. For these reasons SNAP-25 was hypothesized to participate to the pathogenic mechanisms involved in sarcopenia [9]. Reduction of SNAP-25 protein levels has been described in psychiatric patients [39] and is responsible for brain circuits hyperexcitability [12], as well as for learning and cognitive defects in the *SNAP-2*5 heterozygous mice [40]. Reduction of SNAP-25 protein level can be the result of specific gene variants, such as the *SNAP-25* rs363050 polymorphism, which affects SNAP-25 expression either directly [13] or through an epigenetic mechanisms involving specific miRNAs [16]. Here we analyzed the *SNAP-25* rs363050 polymorphism and the expression of 7 circulatory miRNAs, selected because they are differentially expressed in *SNAP-25* heterozygous mice (miR-155-5p, miR-421-3p, miR-451a, miR-425-5p, miR-744-5p, miR-93-5p and mir-495-3p), in sarcopenic patients. Interestingly, bioinformatics analyses showed that the target genes of these 7 miRNAs are involved in pathways relevant for fatty acid metabolism and biosynthesis, and thus in muscle fat accumulation: a risk factor for the development of sarcopenia in old persons [41].

The *SNAP-25* rs363050 (AA) polymorphism was significantly more frequent in sarcopenic patients; interestingly this *SNAP-25* polymorphism was shown to associate with other age-dependent pathologies, including Alzheimer’s disease [34, 42] and Type 2 Diabetes mellitus [43]. Analyses performed on SNAP-25 expression-modulating miRNAs also showed that miR-451a was more expressed in sarcopenic patients, compared to controls. This miRNA is localized in chromosome 17 (17q11.2) and is known to be associated with different human neoplasia, including gastric cancer [44, 45] where it is down-regulated and contributes to tumor formation and progression.

MiR-451a is expressed in skeletal muscle [46], and its expression was shown to be higher in human muscle biopsies of individuals that are low-responders to resistance exercise training [47]. It seems important to underline that this miRNA also plays a critical role in erythrocytes maturation [48]. These results allow to speculate that the increased circulatory level of miR-451a observed in sarcopenic patients could reflect an augmented amount of this miRNA in muscles, or could reflect a compensatory mechanism to induce erythroid maturation, resulting in an increased delivery of oxygen to muscles. Interestingly, a reduction of miR-451a expression was recently described in muscles of non-small cell lung carcinoma patients with cachexia [49], a condition characterized by loss of body weight, systemic inflammation, anorexia, and pronounced loss of skeletal muscle mass [50]. Our analyses were performed in plasma; results confirm the hypothesis that this miRNA can have a role in muscle mass decline diseases. Notably, a statistically significant correlation was detected between the reduction of mir-451a expression and the increase of the change of SPPB score (i.e. the measurement of lower extremity function) after rehabilitation. Importantly, this correlation remains significant even with a multiple logistic regression adjusted with age and frailty index, meaning that this association is not affected by comorbidity and age.

This result suggests that measuring the concentration of this circulatory miRNA might be a novel, easily accessible and non-invasive biomarker to evaluate and monitor the efficacy of rehabilitation programs in sarcopenic patients.

Considering *SNAP-25* rs363050 polymorphisms, the only one miRNA differentially modulated was miR-421-3p, which was observed to be more expressed in AA homozygous sarcopenic patients compared to those with G allele. miR-421-3p is localized in chromosome X (Xq13.2) and was recently suggested to modulate the expression of bone morphogenetic protein-2 (BMP-2), a protein with an important role in the regulation of the differentiation and function of bone and muscle tissue, as well as in muscle regeneration [51]. MiR-451a and miR-421-3p, the two miRNAs that were observed to be differently expressed in sarcopenia, thus regulate two proteins (SNAP-25 and BMP-2) related to muscle function. This result is intriguing and allows to speculate that these two molecules play an important role in muscle trophism and functionality.

All the miRNAs we studied were differentially expressed before and after 30 days of rehabilitation: in particular six miRNAs—miR-155-5p, miR-421-3p, miR-451a, miR-425-5p, miR-744-5p and miR-93-5p—were up-regulated, whereas mir-495-3p was down-regulated.

Although the expression of circulatory miRNAs was already investigated in sarcopenia [52–54], those analyses dealt with muscle-specific miRNAs (myoMir). To our knowledge this is the first study that analyzes the effects of rehabilitative treatment on non-muscle-specific miRNAs expression and modulation in sarcopenic patients. Intriguingly, the concentration of six of these miRNAs in sarcopenic patients before treatment was similar to that observed in healthy controls, suggesting that their modulation is due to rehabilitation and not to pathology, an unexpected result that will need to be further analyzed. To note, miR-155-5p was found to be up-regulated in muscles of elderly people [55] whereas, in accordance with our results, its circulatory expression was seen to be down-regulated in sarcopenic patients compared to controls [53].

Overall, these results could facilitate the identification of biomarkers that could allow the early diagnosis of disease, and might be useful to verify the outcome of rehabilitative/pharmacological therapy in sarcopenia, as well as in other disorders, such as oncological diseases.

A limitation of our study is that the sarcopenia screening was performed just by physical performance indices without imaging parameters that are considered the gold standard to measure the actual ratio of muscle and fat.

## Conclusions

To summarize, these results show the presence of associations between SNAP-25-related genetics and epigenetics and sarcopenia, indicate that non-muscle-related miRNAs are involved in the pathogenesis of this condition, and suggest that the analysis of circulating miRNAs could be used as a predictive biomarker for the outcome of rehabilitation protocols. Further analyses on larger cohorts of patients are needed to validate our results and to better understand the effects of these miRNAs on protein expression and disease development.

## Supplementary Information

**Additional file 1.** Additional table.

## Data Availability

The dataset generated and/or analysed during the current study are not publicy available due to privacy or ethical restriction but are available from the corresponding author on reasonable request.

## Abbreviations

ADHD: Attention deficit hyperactivity disorder
ADL: Activity of daily living
CCI: Charlson Comorbidity Index
CDT: Clock drawing test
ddPCR: Droplet digital polymerase chain reaction
EWGSOP: European Working Group on Sarcopenia in Older People
HC: Healthy controls
HW: Hardy–Weinberg
IADL: Instrument activity of daily living
IQR: Interquartile range
miRNA: MicroRNA
MMSE: Mini-mental state examination
NMJs: Neuromuscular junctions
qPCR: Quantitative polymerase chain reaction
PCR: Polymerase chain reaction
SNAP25: SNARE synaptosomal-associated protein of 25 kDa
SNARE: SNAP receptors
SNP: Single nucleotide polymorphisms
SPPB: Short physical performance battery

## References

[1] Morley JE (2008). Sarcopenia: diagnosis and treatment. J Nut Health Aging.

[2] Cruz-Jentoft AJ, Bahat G, Bauer J, Boirie Y, Bruyere O, Cederholm T, Cooper C, Landi F, Rolland Y, Sayer AA, Schneider SM, Sieber CC, Topinkova E, Vandewoude M, Visser M, Zamboni, M, Writing Group for the European Working Group on Sarcopenia in Older People 2 (EWGSOP2), and the Extended Group for EWGSOP2. Sarcopenia: revised European consensus on definition and diagnosis. Age Ageing 2019;48:16–31.

[3] Liguori I, Russo G, Aran L (2018). Sarcopenia: assessment of disease burden and strategie sto improve outcomes. Clin Interv Aging.

[4] Larsson L, Grimby G, Karlsson J (1979). Muscle strength and speed of movement in relation to age and muscle morphology. J Appl Physiol.

[5] Goodpaster BH, Carlson CL, Visser M, Kelley DE, Scherzinger A, Harris TB, Stamm E, Newman AB (1985). Attenuation of skeletal muscle and strength in the elderly: the Health ABC study. J Appl Physiol.

[6] Shigemoto K, Kubo S, Mori S, Yamada S, Akiyoshi T, Miyazaki T (2010). Muscle weakness and neuromuscular junctions in aging and disease. Geriatr Gerontol Int.

[7] Kwan P (2013). Sarcopenia, a neurogenic syndrome?. J Aging Res..

[8] Ramakrishan N, Drescher MJ, Drescher DG (2012). The SNARE complex in neuronal and sensory cells. Mol Cell Neurosci.

[9] Casati M, Costa AS, Capitanio D, Ponzoni L, Ferri E, Agostini S, Lori E, on behalf of the SA.M.B.A. Project. The biological foundations of sarcopenia: established and promising markers. Front Med. 2019;78:6184. 10.3389/fmed.2019.00184 PMC6700259 31457015

[10] Antonucci F, Corradini I, Fossati G, Tomasoni R, Menna E, Matteoli M (2016). SNAP-25, a known pre-synaptic protein with emerging postsynaptic functions. Front Synaptic Neurosci.

[11] Pozzi D, Corradini I, Matteoli M (2019). The control of neuronal calcium hoemostasis by SNAP-25 and its impact on neurotransmitter release. Neuroscience.

[12] Corradini I, Donzelli A, Antonucci F, Welzl H, Loos M, Martucci R, De Astis S, Pattini L, Inverardi F, Wolfer D, Caleo M, Bozzi Y, Verderio C, Frassoni C, Braida D, Clerici M, Lipp HP, Sala M, Matteoli M (2014). Epileptiform activity and cognitive dieficits in SNAP-25 +/- mice are normalized by antiepileptic drugs. Cereb Cortex.

[13] Braida D, Guerini F, Ponzoni L, Corradini I, De Astis S, Pattini L, Bolognesi E, Benfante R, Fornasari D, Chiappedi M, Ghezzo A, Clerici M, Matteoli M, Sala M. Association between SNAP-25 gene polymorphisms and cognition in autism: functional consequences and potential therapeutic strategies. Transl Psychiatry 2015;5:e500. 10.1038/tp.2014.136 PMC4312830 25629685

[14] Lu TX, Rothenberg ME (2018). MicroRNA. J Allergy Clin Immunol.

[15] Recabarren D, Alarcon M (2017). Gene networks in neurodegenerative disorders. Life Sci.

[16] Agostini S, Mancuso R, Liuzzo G, Bolognesi E, Costa AS, Bianchi A, Clerici M (2019). Serum miRNAs expression and SNAP-25 genotype in Alzheimer's disease. Front Aging Neurosci.

[17] Barr CL, Feng Y, Wigg K, Bloom S, Roberts W, Malone M, Schachar R, Tannock R, Kennedy JL (2000). Identification of DNA variants in the SNAP-25 gene and linkage study of these polymorphisms and attention-deficit hyperactivity disorder. Mol Psychiatry.

[18] Faraone SV, Perlis RH, Doyle AE, Smoller JW, Goralnick JJ, Holmgren MA, Sklar P (2005). Molecular genetics of attention-deficit/hyperactivity disorder. Biol Psychiatry.

[19] Zhang H, Zhu S, Zhu Y, Chen J, Zhang G, Chang H (2011). An association study between SNAP-25 gene and attention-deficit hyperactivity disorder. Eur J Paediatr Neurol.

[20] Hawi Z, Matthews N, Wagner J, Wallace RH, Butler TJ, Vance A, Kent L, Gill M, Bellgrove MA. DNA variation in the SNAP25 gene confers risk to ADHD and is associated with reduced expression in prefrontal cortex. PLoS ONE 2013;8:e60274. 10.1371/journal.pone.0060274 PMC3625226 23593184

[21] Pazvantoğlu O, Güneş S, Karabekiroğlu K, Yeğin Z, Erenkuş Z, Akbaş S, Sarısoy G, Korkmaz IZ, Böke O, Bağcı H, Sahin AR (2013). The relationship between the presence of ADHD and certain candidate gene polymorphisms in a Turkish sample. Gene.

[22] Etain B, Dumaine A, Mathieu F, Chevalier F, Henry C, Kahn JP, Deshommes J, Bellivier F, Leboyer M, Jamain SA (2010). SNAP25 promoter variant is associated with early-onset bipolar disorder and a high expression level in brain. Mol Psychiatry.

[23] Russell AP, Lamon S (2015). Exercise, Skeletal Muscle and Circulating microRNAs. Prog Mol Biol Transl Sci.

[24] Silva GJ, Bye A, El Azzouzi H, Wisloff U (2017). MicroRNAs as important regulators of exercise adaption. Prog Cardiovasc Dis.

[25] Guralnik JM, Simonsick EM, Ferrucci L, Glynn RJ, Berkman LF, Blazer DG, Scherr PA, Wallace RB (1994). A short physical performance battery assessing lower extremity function: association with self-reported disability and prediction of mortality and nursing home admission. J Gerontol.

[26] Petersen RC (2011). Mild cognitive impairment. N Engl J Med.

[27] Shulman KI, Shedletsky R, Silver IL (1986). The challenge of time: Clock-drawing and cognitive function in the elderly. Int J Geriatr Psychiatry.

[28] Yesavage JA, Sheikh JI (2008). Geriatric Depression Scale (GDS) recent evidence and development of a shorter version. Clin Gerontologist.

[29] Testa G, Cacciatore F, Galizia G, Della Morte D, Mazzella F, Russo S, Ferrara N, Rengo F, Abete P (2009). Charlson Comorbidity Index does not predict long-term mortality in elderly subjects with chronic heart failure. Age Ageing.

[30] Katz S, Ford AB, Moskowitz RW, Jackson BA, Jaffe MW. Studies of illness in the aged. The index of BADL: a standardized measure of biological and psychological functions. JAMA 1963;185:914–9. 10.1001/jama.1963.03060120024016 14044222

[31] Lawton MP, Brody EM (1969). Assessment of older people: self-maintaining and instrumental activities of daily living. Gerontologist.

[32] Searle SD, Mitnitski A, Gahbauer EA, Gill TM, Rockwood K (2008). A standard procedure for creating a frailty index. BMC Geriatr.

[33] Washbourne P, Cansino V, Mathews JR, Graham M, Burgoyne RD, Wilson MC (2001). Cysteine residues of SNAP-25 are required for SNARE disassembly and exocytosis, but not for membrane targeting. Biochem J.

[34] Guerini FR, Agliardi C, Sironi M, Arosio B, Calabrese E, Zanzottera M, Bolognesi E, Ricci C, Costa AS, Galimberti D, Griffanti L, Bianchi A, Savazzi F, Mari D, Scarpini E, Baglio F, Nemni R, Clerici M (2014). Possible association between SNAP-25 single nucleotide polymorphisms and alterations of categorical fluency and functional MRI parameters in Alzheimer’s disease. J Alzheimers Dis.

[35] Shah JS, Soon PS, Marsh DJ. Comparison of methodologies to detect low levels of hemolysis in serum for accurate assessment of Serum microRNAs. PLoS ONE 2016;11:e0153200. 10.1371/journal.pone.0153200 PMC4824492 27054342

[36] Lu TP, Lee CY, Tsai MH, Chiu YC, Hsiao CK, Lai LC, Chuang EY. miRSystem: an integrated system for characterizing enriched functions and pathways of microRNA targets. PLoS ONE 2012;7:e42390. 10.1371/journal.pone.0042390 PMC3411648 22870325

[37] Global Burden of Disease Study 2013 Collaborators. Global, regional, and national incidence, prevalence, and years lived with disability for 301 acute and chronic diseases and injuries in 188 countries, 1990–2013: a systematic analysis for the Global Burden of Disease Study 2013. Lancet 2013;386:743–800. 10.1016/S0140-6736(15)60692-4 PMC4561509 26063472

[38] Ziaaldini MM, Marzetti E, Picca A, Murlasitz Z (2017). Biochemical pathways of sarcopenia and their modulation by physical exercise: a narrative review. Front Med (Lausanne).

[39] Najera K, Fagan BM, Thompson PM (2019). SNAP-25 in major psychiatric disorders: a review. Neuroscience.

[40] Fossati G, Morini R, Corradini I, Antonucci F, Trepte P, Edry E, Sharma V, Papale A, Pozzi D, Defilippi P, Meier JC, Brambilla R, Turco E, Rosenblum K, Wanker EE, Ziv NE, Menna E, Matteoli M (2015). Reduced SNAP-25 increases PSD-95 mobility and impairs spine morphogenesis. Cell Death Differ.

[41] Buch A, Carmeli E, Boker LK, Marcus Y, Shefer G, Kis O, Berner Y, Stern N (2016). Muscle function and fat content in relation to sarcopenia, obesity and frailty of old age – An overview. Exp Gerontol.

[42] Guerini FR, Farina E, Costa AS, Baglio F, Saibene FL, Margaritella N, Calabrese E, Zanzottera M, Bolognesi E, Nemni R, Clerici M (2016). ApoE and SNAP-25 polymorphisms predict the outcome of multi dimensional stimulation therapy rehabilitation in Alzheimer's disease. Neurorehabil Neural Repair.

[43] Al-Daghry NM, Costa AS, Alokail MS, Zanzottera M, Alenad AM, Mohammed AK, Clerici M, Guerini FR (2016). Synaptosomal protein of 25KDa (Snap25) polymorphisms associated with glycemic parameters in type 2 diabetes patients. J Diabetes Res.

[44] Pan X, Wang R, Wang ZX (2013). The potential role of miR-451 in cancer diagnosis, prognosis, and therapy. Mol Cancer Ther.

[45] Streleckiene G, Inciuraite R, Juzenas S, Salteniene V, Steponaitiene R, Gyvyte U, Kiudelis G, Leja M, Ruzgys P, Satkauskas S, Kupcinskiene E, Franke S, Thon C, Link A, Kupcinskas J Skieceviciene J. miR-20b and miR-451a are involved in gastric carcinogenesis through the PI3K/AKT/mtor signaling pathway: data from gastric cancer patients, cell lines and ins-gas mouse model. Int J Mol Sci 2019;29:877. 10.3390/ijms21030877 PMC7038213 32013265

[46] Keller P, Vollard NB, Gustafsson T, Gallagher IJ, Sundberg CJ, Rankinen T, Britton SL, Bouchard C, Koch LG, Timmons JA (1985). A transcriptional map of the impact of endurance exercise training on skeletal muscle phenotype. J Appl Physiol.

[47] Davidsen PK, Gallagher IJ, Hartman JW, Tarnopolosky MA, Dela F, Helge JW, Timmons JA, Phillips SM (1985). High responders to resistance exercise training demonstrate differential regulation of skeletal muscle microRNA expression. J Appl Physiol.

[48] Obeidi N, Pourfathollah AA, Soleimani M, Zarif MN, Kouhkan F (2016). The effect of MiR-451 upregulation on erythroid lineage differentiation of murine embryonic stem cells. Cell J.

[49] van de Worp WR, Schols AM, Dingemans AM. Op den Kamp C.M, Degens JH, Kelders MC, Coort S, Woodruff HC, Kratassiouk G, Harel-Bellan A, Theys J, van Helvoort A, Langen RC. Identifcation of microRNAs in skeletal muscle associated with lung cancer cachexia. J Cachexia Sarcopenia Muscle 2020;11:452–63. 10.1002/jcsm.12512 PMC7113505 31828982

[50] Ali S, Garcia JM (2014). Sarcopenia, cachexia and aging: diagnosis, mechanisms and therapeutic options—a mini review. Gerontology.

[51] Ju C, LV Z, Zhang C, Jiao Y. Regulatory effect of miR-421 on humeral fracture and heterotopic ossification in elderly patients. Exp Ther Med 2019;17:1903–11. 10.3892/etm.2019.7146 PMC6364234 30783467

[52] Drummond MJ, McCarthy JJ, Fry CS, Esser KA, Rasmussen BB (2008). Aging differentially affects human skeletal muscle microRNA expression at rest and after an anabolic stimulus of resistance exercise and essential amino acids. Am J Physiol Endrocrinol Metab.

[53] He N, Zhang YL, Zhang Y, Feng B, Zheng Z, Wang D, Zhang S, Guo Q, Ye H (2020). Circulating MicroRNAs in plasma decrease in response to sarcopenia in the elderly. Front Genet.

[54] Iannone F, Montesanto A, Cione E, Crocco P, Caroleo MC, Dato S, Rose G, Passarino G (2020). Expression patterns of muscle-specific miR-133b and miR-206 correlate with nutritional status and sarcopenia. Nutrients.

[55] Jung HJ, Lee KP, Kwon KS, Suh Y (2019). MicroRNAs in skeletal muscle aging: current issues and perspectives. J Gerontol A Biol Sci Med Sci..

